# Lipid nanocarrier containing eugenol for denture hygiene: evaluation of efficacy against *Candida* biofilms

**DOI:** 10.1590/1678-7757-2024-0455

**Published:** 2025-03-10

**Authors:** Irisvaldo Lima GUEDES, Matheus Oliveira do NASCIMENTO, Leandro de Sousa DIAS, Alyne Rodrigues de ARAUJO-NOBRE, Humberto Medeiros BARRETO, Érika de Araújo ABI-CHACRA, Ana Cristina Vasconcelos FIALHO, Gláuber Campos VALE, André Luis Menezes CARVALHO

**Affiliations:** 1 Universidade Federal do Piauí Programa de Pós-Graduação em Odontologia Teresina Piauí Brasil Universidade Federal do Piauí, Programa de Pós-Graduação em Odontologia (PPGO), Teresina, Piauí, Brasil.; 2 Universidade Federal do Delta do Parnaíba Núcleo de Pesquisa de Biodiversidade e Biotecnologia Parnaíba Piauí Brasil Universidade Federal do Delta do Parnaíba, Núcleo de Pesquisa de Biodiversidade e Biotecnologia (Biotec), Parnaíba, Piauí, Brasil.; 3 Universidade Federal do Piauí Departamento de Parasitologia e Microbiologia Teresina Piauí Brasil Universidade Federal do Piauí, Departamento de Parasitologia e Microbiologia, Teresina, Piauí, Brasil.; 4 Universidade Federal do Piauí Programa de Pós-Graduação em Ciências Farmacêuticas Teresina Piauí Brasil Universidade Federal do Piauí, Programa de Pós-Graduação em Ciências Farmacêuticas (PPGCF), Teresina, Piauí, Brasil.

**Keywords:** Eugenol, Nanotechnology, Biofilms, Candida, Acrylic resins

## Abstract

Eugenol has demonstrated efficacy against *Candida* spp., which is highly prevalent in denture wearers. However, the low water solubility and high volatility limit its application. The encapsulation in nanostructured lipid carriers (NLCs) may be a viable approach for developing new sanitizing agents for denture hygiene.

**Objective:**

To develop a sanitizing dispersion for denture hygiene using nanostructured lipid carriers (NLCs) containing eugenol and to evaluate the efficacy against *Candida* spp. biofilms.

**Methodology:**

The formulation was prepared using the ultrasonication method and characterized in terms of particle size (PS), polydispersity index (PDI), zeta potential (ZP), and encapsulation efficiency (EE). The minimum inhibitory concentration (MIC) was determined by the broth microdilution method and the antifungal activity was evaluated by four treatment groups (nanostructured formulation containing eugenol (NFE), free eugenol (FE), saline solution (SS), and the drug-free formulation NFW after eight hours of immersion in biofilms of two Candida species (*Candida albicans* and *Candida glabrata*) adhered to polymethyl methacrylate resin specimens.

**Results:**

The nanoparticles of NFE showed a particle size of 199.5±2.55 nanometers (nm) as measured by DLS, high homogeneity (0.07±0.02), an EE of 83.07±0.23, and a negative ZP (-25.86±0.65). The MICs of FE for *Candida albicans* and *Candida glabrata* were up to 10 times (64 µg/mL) and eight times (128 µg/mL) higher, respectively, than the MICs of NFE (6 µg/mL and 16 µg/mL). The biofilms of these microorganisms showed a significant reduction after immersion in NFE compared to the other tested groups (FE, NBF, and SS) (P<0.0001).

**Conclusion:**

The NFE demonstrated fungicidal activity against the isolated strains and significantly reduced *Candida* biofilms, thus showing promising performance for the sanitization of dentures over eight hours.

## Graphical abstract

**Figure:**
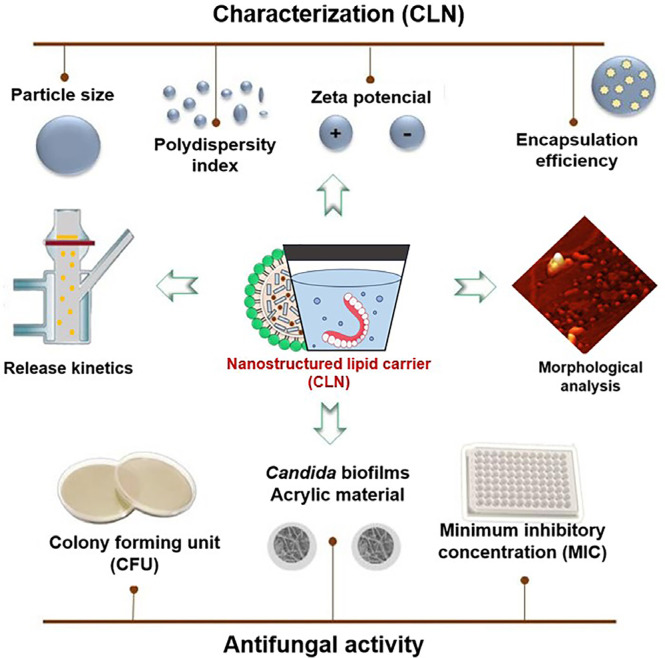
This article is derived from Irisvaldo Lima Guedes's Master's dissertation and is available at the address: https://sigaa.ufpi.br/sigaa/public/programa/noticias_desc.jsf?lc=pt_BR&id=370&noticia=519307121

## Introduction

*Candida spp*. species are responsible for a significant portion of fungal infections in humans.^1^ The most prevalent and pathogenic are *Candida albicans* (*C. albicans*) and *Candida glabrata* (*C. glabrata*), which can trigger infections that compromise individuals’ health.^2,3^ The pathogen negatively impacts users of removable dentures (RD), as it readily adheres to acrylic surfaces. Additionally, the fungus shows polymorphic characteristics, which contribute to the emergence of inflammatory processes in the oral cavity, such as oral candidiasis and prosthetic stomatitis.^4-6^

The hygiene of components of dentures is important for the prevention of oral fungal diseases. The chemical-mechanical method is the most recommended for cleaning and disinfecting dentures.^7^ The most used chemical substances include sodium hypochlorite (SH), chlorhexidine digluconate (CHX), and alkaline peroxides (AP). However, the continued use of these substances can damage the structures of the dentures, alter their chemical and physical properties, and result in high costs.^8 -11^

In this context, the use of new and effective natural products with antibiofilm activity against *Candida* is becoming increasingly promising.^12^ There is proven efficacy of eugenol, the main phenolic component of clove essential oil (70-90%), against *Candida* strains. However, it is volatile and has limited solubility and dose-dependent toxicity.^13,14^

Nanostructured lipid carriers (NLCs) are part of a binary pharmaceutical system composed of solid and liquid lipids that enable the retention of lipophilic actives, protecting them from degradation and improving their bioavailability due to their ability to modulate release.^15,16^ The use of such systems makes isolated compounds such as eugenol even more promising for the development of antifungal sanitizing products.^17^

To date, no lipid nanocarrier containing encapsulated eugenol has been developed as a viable alternative for the hygiene of removable dentures (RDs). Therefore, this study aimed to develop a nanostructured lipid carrier dispersion containing eugenol, characterize it, and evaluate the activity against *Candida* biofilms adhered to an acrylic material used in RDs.

## Methodology

### Experimental design

This is an *in vitro* laboratory study*.* The formulation was developed, characterized, and tested to assess its potential antifungal effect on isolates and biofilms of two species of *Candida* (*C. albicans* and *C. glabrata*) that adhered to the surfaces of specimens of a heat-cured acrylic resin (RAT). These were polished and sterilized before the experiment. The number of specimens that were used in the study was determined based on a pilot study and a sample size of three or more was found to provide a good degree of reproducibility.

### Preparation of the nanostructured formulation containing eugenol (NFE) and nanostructured formulation without eugenol (NFW)

The emulsification method followed by ultrasonication was used.^18^ The formulation consisted of a solid lipid (carnauba wax (Lot: 0210701/2022)), liquid lipid (oleic acid (Synth, Diadema, Brazil)), aqueous surfactant solution (poloxamer 407^®^ prepared at 5% (ChemSpecs, São Paulo, Brazil)) and eugenol (Biodinâmica, Ibiporã, Brazil)) at concentrations of 7%, 3%, 89.7% and 0.3%, respectively. All the components, except eugenol, were heated to 95 ºC (10 ºC above the melting point of the solid lipid (85 ºC)).^18^ Homogenization was then performed using a macro ultrasonic probe sonicator (Eco-sonics, Indaiatuba, Brazil) set to a frequency of 20 kHz, an amplitude of 80 µm, and a power level of 70% for 10 minutes. Subsequently, the concentration of eugenol in the formulation was quantified via UV-VIS spectroscopy. The NFW was prepared following the same parameters as the NFE but without the incorporation of eugenol.

### Characterization of the NFE

#### Particle size (PS), polydispersity index (PDI), and zeta potential (ZP)

The particle size (TP) and polydispersity index (PDI) were determined using the dynamic light scattering (DLS) technique, while the zeta potential (PZ) was measured via electrophoretic light scattering. The analyses were conducted using the Zetasizer NanoZS90 (Malvern Panalytical, Gondomar, Portugal) with a fixed detection angle of 90º, a resolution of 0.6 nanometers (nm), and sensitivity across a wide range of sizes (0.6 nm to 6 µm). The measurements were performed at a temperature of 25 ºC, with a measurement time of 60 to 120 seconds for each analysis. Deionized water was used as the solvent for sample dilution. Measurements were performed in triplicate.

#### Encapsulation efficiency (EE) of eugenol

The method used by Vijayakumar, et al.^19^ (2017) and Lopes, et al.^20^ (2017) was employed to determine the drug encapsulation efficiency (EE). To verify the amount of free eugenol (EL), the NFE was centrifuged using an ultracentrifugation filter (Millipore, Darmstadt, Germany). Subsequently, the quantification of EL was performed using a UV-VIS spectrophotometer (Shimadzu, Kyoto, Japan) at a wavelength of wavelength of 291.4 nm (first-order derivative). The content (ET) was determined by reading the second dilution in the UV-VIS spectrophotometer. The analysis was conducted in triplicate. The amount of encapsulated active ingredient was determined using the formula: EE = (ET - EL) / ET × 100.

## Evaluation of morphology by atomic force microscope (AFM)

The formulations containing eugenol (NFE) and without eugenol (NFW) were prepared by depositing a volume of 20 μL of the diluted nanoformulation at a ratio of 1:100 (in ultrapure water) onto a freshly cleaved mica surface at room temperature. After a drying period of 24 hours, analysis was performed using a TT-AFM model (Workshop, United States) in tapping mode, with silicon probes (TAP300-G, Ted Pella) and a resonance frequency of approximately 240 kHz. The images (512×512 pixels) were analyzed using Gwyddion 2.60 software, and the average size of the nanoparticles was expressed as the mean ± standard deviation (SD).^2 1^

## *In vitro* release kinetics

The release drug substance was investigated using Franz diffusion cells, with a diffusion area of 1.15 cm^2^. The receptor medium was prepared using a phosphate buffer solution and ethyl alcohol (Êxodo científica, Sumaré, Brazil) (8:2, pH 7.4),^22^ and a dialysis membrane (Spectra/Por^®^ Dialysis Membrane, MWCO 3500, Spectrum Laboratories Inc., USA) was used to separate the donor compartment from the receptor compartment and was prepared via an initial hydration using distilled water at 25 ºC for 30 minutes. Subsequently, the medium was rinsed to ensure the complete removal of impurities. The membrane was then immersed in a receptor medium for 24 hours prior to the start of the experiment to minimize variations during diffusion.

Two groups were prepared: test solutions (NFE (0.5 g)) and free eugenol solution (0.5 g). Six Franz cells were prepared, each containing 14 mL of the receptor medium for triplicate analysis. The temperature of the release medium was controlled at 37±0.5°C, and the magnetic stirring speed (SPLabor, São Paulo, Brazil) was set at 300 rpm. At time intervals of 0, 30 min, 1 h, 2 h, 4 h, 6 h, 8 h, 10 h, and 24 h, 3 mL of the release medium was collected from each cell. The amount of released eugenol was quantified by reading the samples using a UV-VIS spectrophotometer.

## Antifungal activity of NFE

### Determination of the minimum inhibitory concentration (MIC) and the minimum fungicidal concentration (MFC).

The microorganisms that were used are part of the microbiological collection of the Microbiology Research Laboratory at the Federal University of Piauí, where they are maintained on solid culture medium Sabouraud Dextrose Agar/Chloramphenicol Neogen (Kasvi, São José dos Pinhais, Brazil) at 8 ºC. The standard strains *C. albicans* ATCC 10231 and *C. glabrata* ATCC 2001 were inoculated and incubated in an oven at 37 °C in Brain Heart Infusion (BHI) medium (Kasvi, São José dos Pinhais, Brazil) at 3%. The optical density of the fungal suspensions was adjusted to be equivalent to 0.5 on the McFarland scale, corresponding to an approximate concentration of 1×10⁶ to 5×10⁶ colony-forming units (CFU)/mL. The adjustment was made using a spectrophotometer (Bel Photonics SP – 2000 UV, Piracicaba, Brazil), with absorbance measured at a wavelength of 530 nm.

The determination of the minimum inhibitory concentration (MIC) was evaluated using the broth microdilution method according to Leal, et al.^23^ (2019). The determination of the minimum fungicidal concentration (MFC) was performed using the broth microdilution method, confirmed by the absence of growth on solid Sabouraud Dextrose Agar. To differentiate fungicidal from fungistatic activity, the MFC was equal to or up to four times greater than the minimum inhibitory concentration (MIC). The values were compared between free eugenol and the test formulations, with and without eugenol, ensuring methodological rigor and reproducibility. Analyses were conducted in triplicate, and the results were expressed as the geometric mean.

## Pre-formed *Candida* biofilms

### Preparing and randomizing the specimens

The test specimens were fabricated with a thickness of 2 mm and a diameter of 12 mm in a circular shape using thermopolymerizable polymethyl methacrylate (PMMA) acrylic resin (Vipicril Plus clear, Florianópolis, Brazil). The finishing surface was performed with a polisher (Arotec, Cotia, Brazil) that was adapted with abrasive sanding discs (Sait, Guarulhos, Brazil) (grit sizes 600 and 1200). Polishing was conducted using acrylic polishers (brown, green, and yellow abrasive points (Exa-Technique, São Paulo, Brazil)). Simple randomization was used to allocate the test specimens into their respective pre-established groups. As a result, a randomly generated sequence was employed, using a table of random numbers corresponding to the groups.

## Determining the treatment groups

Four comparison groups were used to evaluate the anti-biofilm activity of *C. albicans* and *C. glabrata* in 32 test specimens. Table 1 describes the sample division according to the treatment groups.

**Table 1:** Division of the specimens for the application of the treatment groups.

Groups	Treatments	Number of specimens	
		*C. albicans*	*C. glabrata*
Intervention	NFE	4	4
Intervention without active ingredient	NFW	4	4
Positive control	FE 0.3%	4	4
Negative control	SS 0,9%	4	4
Total	4	32	

NFE (Nanostructured formulation containing eugenol); NFW (Nanostructured formulation without eugenol); FE (Free eugenol); SS (saline solution).

## Biofilm formation methodology and treatment application

Standard strains of *C. albicans* ATCC 10231 and *C. glabrata* ATCC 2001 were used. Sabouraud Dextrose Agar with Chloramphenicol (Difco Laboratories) was used to reactivate and maintain the strains. To promote biofilm growth, 30 mL of Yeast Peptone Dextrose (YPD) broth was used, containing yeast extract (10 g/L; Isofar, Duque de Caxias, Brazil), dextrose (20 g/L; Dinâmica, Indaiatuba, Brazil), and peptone (20 g/L; Becton Dickinson, East Rutherford, United States). All media were prepared following the manufacturer’s descriptions.

For biofilm formation, yeast-like cells were seeded in Sabouraud Dextrose Agar (SDA) (Difco Laboratories) and inoculated into 30 mL of Yeast Peptone Dextrose (YPD) broth. They were incubated at 37°C for 18 hours in a BOD incubator (7Lab, Rio de Janeiro, Brazil). Then, part of the suspension was transferred to a sterile YPD medium to readjust the cell concentration to 10^6^cells/mL, according to an optical density (OD) of 2.0 on the McFarland scale.

In the adhesion phase evaluation, 0.5 mL of the standardized cell suspension was transferred to a 24-well plate (Kasvi, São José dos Pinhais, Brazil) containing an acrylic resin specimen at the bottom of each well. Initial adhesion was conducted by incubating the plate in the incubator for six hours at a temperature of 37°C. After this period, the contaminated suspension was removed, and a new aliquot of 0.5 mL of YPD medium was added to each well, which remained for an additional 18 hours under the same conditions. After this period, the medium was again removed, and 0.5 mL of each treatment was added to each well. The plate was then incubated at 37°C for eight hours.

To assess the antifungal activity of the treatments, the specimens were transferred to a new plate with wells containing 0.5 mL of saline solution after 24 hours of biofilm development. Biofilm was removed from the solution by rigorous pipetting. Approximately 500 µL of the obtained suspension was transferred to sterile Eppendorf (Kasvi, São José dos Pinhais, Brazil). From this suspension, 100 µL were aliquoted to perform serial dilutions (10⁻^1^ to 10⁻⁷) in Eppendorf tubes containing 900 µL of 0.9% saline solution. Each dilution was plated on ASD agar in quadruplicate and incubated for 48 hours at 37°C for subsequent counting of colony-forming units (CFUs).

## Sterility and contamination control

All experimental procedures were conducted in a controlled environment, using laminar flow hoods and pre-disinfected surfaces. Materials and reagents were sterilized by autoclaving. Stringent aseptic techniques were employed, including the use of appropriate personal protective equipment.

## Statistical analysis

The assumptions of variance equality and normal distribution of errors were checked for all tested response variables. The original CFU data were transformed into base 10 logarithms. The Graphpad Prism 9.02 software (Graphpad, La Jolla, CA, USA) was used for statistical analysis. The SHAPIRO-WILK’s test was conducted to assess the normality of the data distribution. Since the data showed a normal distribution, an analysis of variance (ANOVA) was applied, followed by Tukey’s test for multiple comparisons. These tests were chosen because they satisfy the statistical assumptions of the transformed data and provide robust and reliable analyses for comparing variables between groups. This approach is consistent with widely accepted statistical analysis used in experimental studies. The significance level was set at 5%.

## Results

### Characterization of the NFE

#### TP, IPD, PZ, and EE

The nanoparticles of NFE and NFW showed nanometric size, high homogeneity, and negative EE and ZP (Table 2). There were no significant changes in the parameters in the presence of the drug in the formulation (P>0.05).

**Table 2:** Physico-chemical characterization of the formulations (NFE and NFW).

Parameters	NFE	NFW
	**Mean ± SD**	**Mean ± SD**
PS (nm)	199.5±2.55	198.16±3.70
PDI	0.07±0.02	0.09±0.04
ZP (mV)	- 25.86±0.65	-24.33±0.23
EE (%)	83.07± 0.23	-

Legend: mV (millivolt); nm (nanometers); SD (standard deviation); NFE (Nanostructured formulation containing eugenol); NFW (Nanostructured formulation without eugenol); PS (Particle size); PDI (Polydispersity index); ZP (Zeta potential); EE (Encapsulation efficiency).

## Morphology determination by AFM

**Figure 1:**
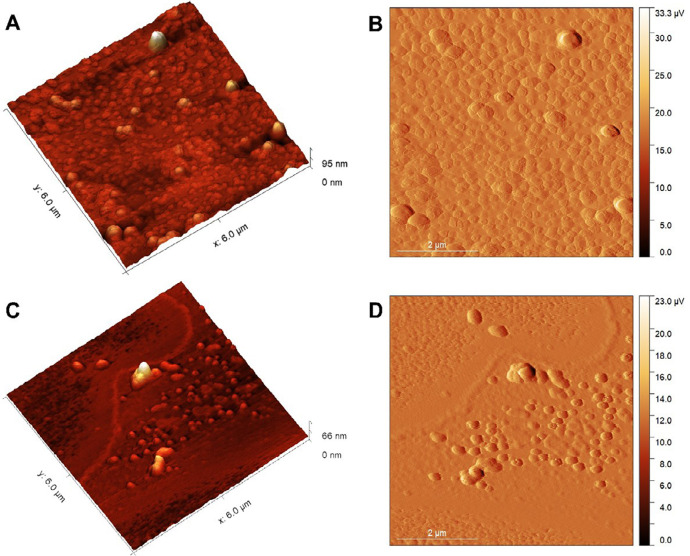
AFM images. µV- microvolt ; 3D topography images (A and C) and 2D amplitude images (B and D). NFW (A and B); NFE (C and D). Scale = 2 µm (micrometer).

**Figure 2:**
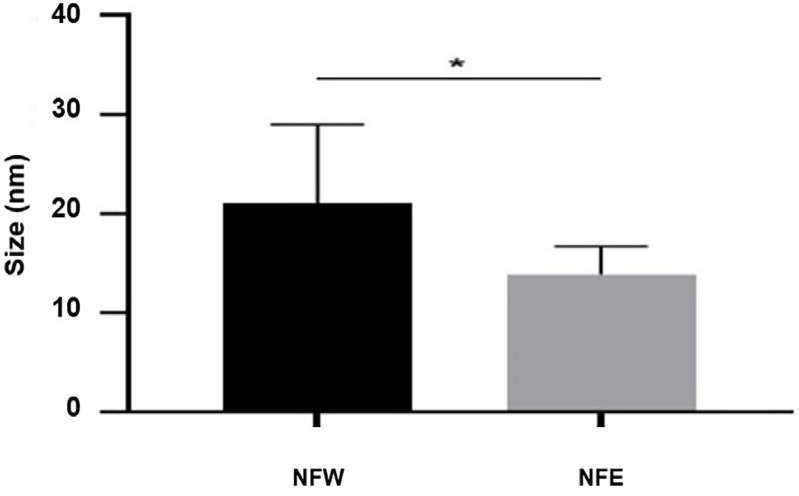
Nanoparticle size graph from AFM analysis. *p<0.0001 after Mann-Whitney test. NFE (Nanostructured formulation containing eugenol); NFW (Nanostructured formulation without eugenol); nm (nanometer).

Figure 1 shows nanoparticles can be observed using Atomic Force Microscopy (AFM). The images reveal spherical nanoparticles in both analyzed samples, consistent with the extracted profile. The average size for NFW was 21.04±7.92 nm, whereas for NFE was 13.91±2.79 nm (Figure 2).

## *In vitro* eugenol release kinetics

**Figure 3:**
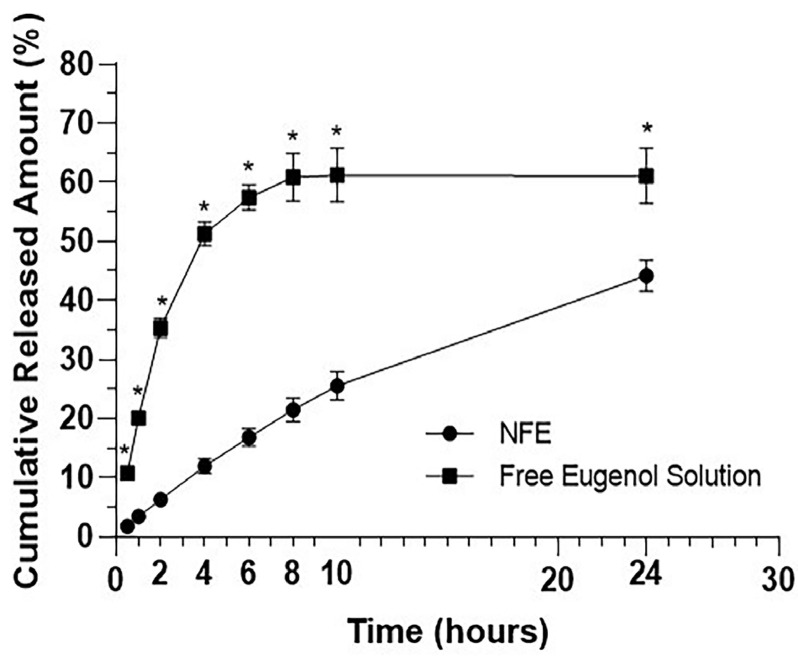
*In vitro* release profile of eugenol encapsulated in nano-structured lipid carriers and free eugenol solution. *P<0.05 after two-way ANOVA test. NFE (Nanostructured formulation containing eugenol).

The cumulative amounts released (over 24 hours) from NFE and the free eugenol solution were 44.21% and 61.11%, respectively (Table 3). NFE showed a controlled release profile throughout the entire kinetic profile. The concentrations of the released free eugenol were significantly higher (P<0.05) at all collection points from the free eugenol solution compared to the concentrations that were released from NFE (Figure 3).

**Table 3:** Inhibitory effect of nanostructured formulations (NFE and NFW) and eugenol against Candida strains.

Formulations	*Candida* species	(μg/mL)			
		MIC	MFC	MFC/MIC	Inhibitory effect
NFW	*C. albicans* ATCC 10231	≥1024	≥1024	-	No activity
	*C. glabrata* ATCC 2001	≥1024	≥1024	-	No activity
Eugenol	*C. albicans* ATCC 10231	64	64	01:01	Fungicide
	*C. glabrata* ATCC 2001	128	128	01:01	Fungicide
NFE	*C. albicans* ATCC 10231	6	6	01:01	Fungicide
	*C. glabrata* ATCC 2001	16	16	01:01	Fungicide

## Microbiological analysis of CIM and CFM

The MICs of eugenol against the *C. albicans* and *C. glabrata* strains were 64 and 128 μg/mL, respectively. NFE reduced the values MIC by 10 and eight times (6 and 16 μg/mL, respectively). In addition, the MFCs were equal to the MIC values for both *Candida* strains in both solutions. The NFE showed fungicidal action against both test strains at the MIC values (Table 3).

## Antifungal activity of NFE on *Candid*a biofilms

**Figure 4:**
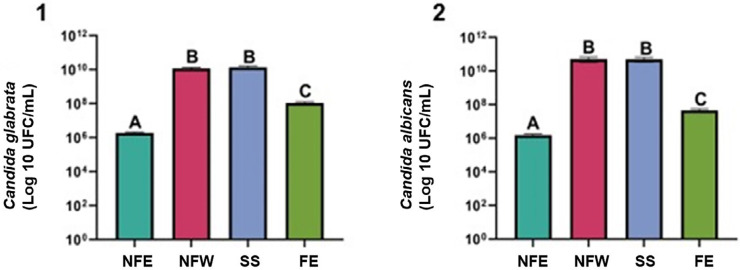
Activity of the test solutions on *C. albicans* and *C. glabrata* biofilms. One-way ANOVA and Tukey test: NFE vs. NFW (P˂0.0001); NFE vs. SS(P˂0.0001); NFE vs. FE (P˂0.0001); NFW vs. SS (P˃0.999) NFW vs. FE (P˂0.0001); FE vs. SS (P˂0.0001). NFE (Nanostructured formulation containing eugenol); NFW (Nanostructured formulation without eugenol); FE (Free eugenol); SS (saline solution).

Quantitative analysis of biofilm by viable cell count (expressed as colony forming units (CFU)) revealed that the NFE showed the highest antimicrobial activity, with a significant reduction of biofilms compared to the other groups (p<0.0001) (Figure 4 (1 and 2)). Free eugenol (FE), even at the same concentration as the NFE (0.3%), showed inferior efficacy against the biofilms of *C. albicans* and *C. glabrata* (p<0.0001). The NFW and the saline solution (SS) showed no significant difference in reducing biofilms (p>0.999) (Figure 4 (1 and 2)).

## Discussion

A power of 70% and a stirring time of 10 minutes were selected to maximize emulsification efficiency and system stability, while preserving the integrity of the active component and ensuring compatibility, as reported by Fang and Bhandari^24^ (2010), Silva, et al.^25^ (2011), and Bolequi, et al.^26^ (2016). The nanoparticles of the FNE showed nanometric sizes (199.5±2.55 nm as analyzed using the Zetasizer (Table 2) and 13.91±2.79 nm using atomic force microscopy (AFM) (Figure 1)). Particles are considered nanoparticles when their size falls within the nanometric scale, ranging from 1 nm to 1000 nm. The main advantage is that physicochemical and functional properties improve as they transition to the nanoscale.^27^ Additionally, the reduction in average diameter can enhance the stability of lipid nanoparticles, facilitating efficient target delivery and preventing rapid drug elimination.^28^

There was a reduction in particle size in the atomic force microscopy (AFM) readings compared to the Zetasizer measurements. This can be explained by the differences in the methods and precision levels of the analysis. The Zetasizer technique measures dynamic light scattering, which is used to calculate the average diameter rather than the size of the particles.^29,30^ In contrast, AFM evaluates three-dimensional information in real-time about lipid systems, with a resolution close to one nanometer.^31,32^ Therefore, it provides a more accurate nanometric analysis of the nanoparticles.

The polydispersity index (PDI) refers to the degree of non-uniformity in a particle size distribution.^33^ The PDI value ranges from 0.0 (for a perfectly homogeneous sample regarding particle size) to 1.0 (for a highly heterogeneous sample with multiple particle size populations). Values that are less than or equal to 0.2 are generally more acceptable for optimizing nanoparticles containing polymers. According to Tamjidi, et al.^32^ (2013), values above 0.5 indicate a very broad particle size distribution, characterizing high instability that leads to unpredictable behavior and reduces the efficacy of the bioactive system. In this study, monodisperse nanoparticles were obtained (0.07±0.02; Table 2), indicating acceptable stability.

ZP values above +30 mV or below -30 mV are considered ideal for colloidal dispersions to maintain good stability.^34,35^ This study’s results showed a negative ZP of -25.86±0.65 (Table 2). Although these values are below the average threshold, dispersion can remain stable. This stability can be attributed to the presence of the steric stabilizer Poloxamer 407. Surfactants such as Poloxamer, when adsorbed onto the particle surface, alter the particle’s shear plane.^36^ Its polymeric chain promotes particle repulsion (entropic forces), maintaining a considerable distance between them.

The high encapsulation efficiency obtained (83.07%±0.23, Table 2) can be attributed to the presence of the liquid lipid in the formulation, as it allows for the imperfect formation of the lipid matrix and, consequently, enhances the drug entrapment.^37^ Eugenol shows high solubility in the oily phase, which can also be a contributing factor to the improved encapsulation efficiency.^38^ Studies indicate that active substances with high lipid solubility tend to show relatively high encapsulation efficiencies, typically above 80%.^39^

The NFE showed a controlled release profile with a cumulative amount of eugenol of 44.21% in 24 hours. This was expected due to the system’s ability to encapsulate an active ingredient within a disordered lipid matrix, which hinders rapid release.^37,40^ The type of stabilizer can also influence release control. Sulfactants such as poloxamer 407, which was used in this study, contribute to a slower degradation of the system due to their steric effect.^41^

NFE and FE demonstrated fungicidal activity against isolated *Candida* strains (Table 3) and in *Candida* biofilms (*C. albicans* and *C. glabrata*) (Figure 4). Several studies have confirmed the antifungal action of eugenol against this microorganism species.^12, 17,42,43^ Regarding the action mechanism, this active compound can bind to the *Candida* membrane and decrease ergosterol biosynthesis due to its ability to interact with the fungal membrane and damage its cell wall.^44^ Additionally, eugenol can increase levels of lipid peroxidation and reactive oxygen species, thereby inducing oxidative stress that leads to increased permeability of the fungal cell membrane.^45,46^ This drug substance may also interact with adhesive proteins, such as ALS, resulting in a considerable decrease in the fungus’s adhesion capacity and promoting the disruption of formed biofilms (*C.albicans*).^3^

The MIC (6 µg/mL) and MFC (6 µg/mL) of NFE against *C. albicans* strains were reduced by up to 10 times compared to the concentrations of FE (MIC (64 µg/mL) and MFC (64 µg/mL)) (Table 3). In addition, there was a significant reduction in pre-formed biofilms after application of NFE for eight hours compared to FE, even though both contained the same concentration (0.3%). These results can be justified by the presentation of FNE particles at the nanoscale (13.91 nm), which enables an increased surface area of contact and consequently enhances the chances of the nano-encapsulated active ingredient coming into contact with the fungal cell wall in the medium.^18^ Furthermore, the system is capable of controlling eugenol release, thereby increasing its activity and enabling targeted action against the microorganism.^47,48^

Nanoencapsulation promotes specific molecular interactions that enhance the antifungal mechanisms of eugenol. For instance, encapsulation facilitates the incorporation of eugenol into the fungal cell membrane, strengthening its binding to ergosterol and compromising fungal membrane integrity.^45^

Additionally, the targeted delivery of encapsulated eugenol enhances its interaction with ALS adhesive proteins, significantly reducing adhesion and disrupting the biofilm structure.^49^ These mechanisms explain the superior efficacy of the NFE compared to free eugenol, even at equivalent concentrations, in combating *Candida* biofilms.

### Limitations of the study

Most tests were conducted in controlled laboratory settings (in vitro), which may not fully replicate the actual conditions of the oral cavity, such as the presence of saliva, pH variations, temperature fluctuations, and the complete oral microbiome. Although the tests demonstrated the efficacy of the NFE, it is crucial to assess its long-term effects. Prolonged use of the formulation over months or years may reveal factors such as microbial resistance or potential cumulative effects on acrylic materials.

The comparison was made using biofilms that were formed on specimens of heat-polymerized acrylic resin, which simulate dental prostheses. However, these conditions may not accurately reflect biofilm formation on real dentures used by patients, as they are influenced by individual factors such as oral hygiene, diet, and overall health. Furthermore, the formulation compatibility with different types of denture materials — not only acrylic resin — and metallic components, such as cobalt-chromium or titanium clasps in partial dentures, is yet to be investigated. Different materials may show varying reactions to formulation. These limitations highlight areas for future research and underscore that, despite the promising results, the practical application and generalizability of the findings require validation in a broader context.

## Conclusions

The nano-structured formulation loaded with eugenol was successfully developed and showed characterization parameters within acceptable values. Furthermore, it showed fungicidal activity against isolated *Candida* strains and significantly reduced the biofilms of *Candida* (*C. albicans* and *C. glabrata*). The NFE, containing 0.3% eugenol, demonstrated superior performance compared to free eugenol (FE) at the same concentration. Therefore, lipid nanocarriers (LNCs) demonstrated significant potential for administering eugenol in the hygiene of dental prostheses, offering promising prospects for future applications in dentistry
